# Biological sex and transfusion practices in the NICU

**DOI:** 10.1038/s41390-025-04328-y

**Published:** 2025-08-05

**Authors:** Krithika Lingappan

**Affiliations:** Pediatrics, Children’s Hospital of Philadelphia, Philadelphia, PA USA

Extremely premature infants frequently require packed red blood cell (pRBC) transfusions due to the high risk of anemia. Prior research has linked pRBC transfusions to increased pro-inflammatory cytokines and adverse neurodevelopmental outcomes and suggested potential sex-specific differences in inflammatory responses to transfusions.^1–4^ The study by German et al. aimed to explore this potential sex-specific effect and its implications for neurodevelopment.^5^ The study showed that a heavier transfusion burden is linked to worse developmental scores in early childhood for both sexes, reinforcing the importance of judicious transfusion practices for all extremely preterm infants.

Previously, it had been postulated that neurodevelopmental outcomes associated with different transfusion thresholds may have a sex-specific effect. Nopoulous et al. reported that preterm females with higher hemoglobin thresholds (liberal transfusions) exhibited the greatest degree of structural brain abnormality. A follow-up study revealed that females in the liberal group also had lower verbal fluency.^1,3^ Follow-up studies from the TOP trial^6^ have indicated that in male preterm infants, higher pre-transfusion hemoglobin values (more liberal transfusions) were associated with higher cerebral white matter volume and BSID-III scores.^7,8^ This led the authors to postulate that providers may need to consider sex as a biological variable when deciding transfusion thresholds in premature neonates.

In this study, German et al. present the post-hoc analysis of the Preterm Erythropoietin Neuroprotection (PENUT) Trial, focusing on the neurodevelopmental outcomes of extremely preterm infants after packed red blood cell (pRBC) transfusion, with a specific examination of sex-based differences. There were two primary study questions: whether infant sex affected the cytokine response to transfusions? and whether there was an interaction by sex with post-transfusion cytokine levels, magnetic resonance imaging (MRI) scores at 36 weeks PMA, and 2-year outcomes assessed by composite scores on the Bayley Scales of Infant Development, third edition (BSID-III). Of note, for these analyses three study subsets analyzed: Biomarker cohort (cytokines at 0–7 and 7–14 days): 182 infants (87 female, 95 male), MRI cohort (at 36 weeks PMA): 220 infants (110 female, 110 male), and the neurodevelopmental assessment cohort (BSID-III at 24 months corrected age): 692 infants (338 female, 354 male). The study team used Generalized Estimating Equation (GEE) models to examine associations, including interaction terms between infant sex and transfusion volume, number of transfusions, or lowest Hct. Models were adjusted for various confounding factors (e.g., gestational age, erythropoietin treatment, chorioamnionitis, 5 min Apgar score, severe sepsis, necrotizing enterocolitis, spontaneous intestinal perforation, severe intraventricular hemorrhage, severe bronchopulmonary dysplasia, other transfusions, and maternal education).

Male infants had higher IL-6 levels with increasing transfusion volume, suggesting a greater inflammatory response in males following transfusion in the second week after birth. This finding contrasts with a smaller, single-center analysis by Benavides et al.^4^ (a sub-study of the TOP trial), who reported higher IL-6 levels in females. The Benavides study also reported a sex-specific variation in other inflammatory markers in response to red blood cell transfusions during the neonatal period. They reported an increase in MCP-1 levels exclusively in female infants. They also reported that higher concentrations of MCP-1 were associated with worse Bayley-III cognitive and motor scores (in both male and female infants). It is important to note that in the Benavides study, cytokine analysis was performed in 71 infants, and neurodevelopmental assessment was performed in 26 infants. So, the sample size was smaller compared to the present study. MCP-1 levels were not measured in the German et al. study.

Importantly, in the German et al. study, the short-term effects on cytokine levels did not translate to long-term outcomes as assessed by the MRI findings (Kidokoro score) and BSID-III scores. The authors show that the total pRBC transfusion volume was significantly negatively associated with all BSID-III subscales (Motor, Cognitive, Language) across both sexes. While males had generally lower neurodevelopmental scores, their response to transfusions in terms of long-term outcomes does not differ significantly when compared to females. The authors also performed additional sensitivity analyses using the number of transfusions instead of volume or focusing only on the placebo group. These analyses did not meaningfully alter the findings. The findings highlight the importance of minimizing transfusions in preterm infants due to the potential adverse effects of transfusions on long-term outcomes

Other studies have suggested sex-linked factors in transfusion outcomes. For example, a large multi-center study by Patel et al.^9,10^ reported improved outcomes in very low birth weight infants who received blood from female donors, implying that either donor sex or recipient sex can modulate transfusion-related inflammation.

Neither the European ETTNO trial^11^ nor the NICHD TOP trial^6^ found significant differences in 2-year neurodevelopment between liberal vs. restrictive transfusion strategies, suggesting that simply using a higher or lower hemoglobin threshold did not dramatically alter brain outcomes. The authors in the German et al. study speculate that the magnitude of transfusion exposure may be a key factor: in controlled trials, the difference in cumulative blood volume transfused between study arms (restrictive and liberal) was relatively small (e.g. only ~21 mL difference on average in the ETTNO trial), whereas in the PENUT cohort some infants received very high volumes (over 300 mL) during the NICU hospitalization. This greater exposure range could make the transfusion effects on the brain more detectable in observational studies. Sicker babies usually receive a higher number of transfusions. Even so, after adjusting for illness severity, the present study still found no sex-modification of MRI outcomes, suggesting any transfusion-related brain injury risk is similar for males and females in current practice.

The authors also analyzed the association between the lowest Hct and outcomes. Generally, infants with lower minimum Hct had lower BSID-III scores, though this was not statistically significant across all domains. A non-significant trend suggested that males might be more sensitive to the effects of anemia on motor and cognitive scores, which was postulated by prior studies.^7,8^

A significant negative association was reported between the total volume of pRBC transfusions and long-term neurodevelopmental outcomes (BSID-III scores) across all domains, cognitive, language, and motor, regardless of sex, at 24 months corrected age, with greater transfusion exposure correlating with lower scores on each subscale. The negative association persisted even after accounting for the degree of anemia (hematocrit levels) and other indicators of illness severity in the regression models. These findings reinforce a growing body of evidence linking a higher number of transfusions to adverse neurodevelopment. It should be noted, however, that while the association is robust, this study design cannot prove causation—infants requiring many transfusions may be sicker or more immature to begin with. The authors did attempt to adjust for baseline risk factors, and the transfusion effect remained significant, though residual confounding by illness severity may still play a role.

In summary, total transfusion burden remained the strongest predictor of worse MRI and BSID-III outcomes at two years, regardless of sex. Thus, while further research may clarify mechanistic underpinnings, the evidence from the German et al. study does not support sex-specific transfusion thresholds. Clinicians should continue to minimize unnecessary transfusions and optimize anemia management for all extremely preterm infants.
